# Polysaccharide from *Smilax glabra* Roxb Mitigates Intestinal Mucosal Damage by Therapeutically Restoring the Interactions between Gut Microbiota and Innate Immune Functions

**DOI:** 10.3390/nu15194102

**Published:** 2023-09-22

**Authors:** Muhammad Abaidullah, Shaokai La, Mengqi Liu, Boshuai Liu, Yalei Cui, Zhichang Wang, Hao Sun, Sen Ma, Yinghua Shi

**Affiliations:** 1 College of Animal Science and Technology, Henan Agricultural University, Zhengzhou 450002, China; drabaidullah@henau.edu.cn (M.A.); lskmymailbox@163.com (S.L.); 2019110376@sdau.edu.cn (M.L.); boshuailiu@126.com (B.L.); yaleicui423@henau.edu.cn (Y.C.); zcwang@henau.edu.cn (Z.W.); sunhao@henau.edu.cn (H.S.); ms0321021@163.com (S.M.); 2 Henan Key Laboratory of Innovation and Utilization of Grassland Resources, Zhengzhou 450002, China; 3 Henan Forage Engineering Technology Research Center, Zhengzhou 450002, China

**Keywords:** *Smilax glabra* Roxb polysaccharides, ulcerative colitis, goblet cells, tight junction proteins, cytokines, microbiota

## Abstract

*Smilax glabra* Roxb (*S. glabra*) is a conventional Chinese medicine that is mainly used for the reliability of inflammation. However, bioactive polysaccharides from *S. glabra* (SGPs) have not been thoroughly investigated. Here, we demonstrate for the first time that SGPs preserve the integrity of the gut epithelial layer and protect against intestinal mucosal injury induced by dextran sulfate sodium. Mechanistically, SGPs mitigated colonic mucosal injury by restoring the association between the gut flora and innate immune functions. In particular, SGPs increased the number of goblet cells, reduced the proportion of apoptotic cells, improved the differentiation of gut tight junction proteins, and enhanced mucin production in the gut epithelial layer. Moreover, SGPs endorsed the propagation of probiotic bacteria, including *Lachnospiraceae bacterium*, which strongly correlated with decreased pro-inflammatory cytokines via the blocking of the TLR-4 NF-κB and MyD88 pathways. Overall, our study establishes a novel use of SGPs for the treatment of inflammatory bowel disease (IBD)-associated mucosal injury and provides a basis for understanding the therapeutic effects of natural polysaccharides from the perspective of symbiotic associations between host innate immune mechanisms and the gut microbiome.

## 1. Introduction

Ulcerative colitis (UC) is an inflammatory bowel disease (IBD) that affects the colonic mucosa and submucosa. The onset and progression of UC are multifaceted and are associated with a combination of genetic, microbial, and environmental factors [1,2,3,4]. Individuals with UC mostly manifest with hematochezia, diarrhea, weight loss, and abdominal pain [5,6]. The current spectrum of therapeutic options includes amino salicylic acids (ASA), immunosuppressants, glucocorticoids, and incipient biological remedies, which typically include anti-tumor necrosis factor (TNF) biological therapies, anti-TNF agents, anti-adhesion agents, pro-inflammatory cytokine inhibitors, and downstream signaling inhibitors [7]. Although these drugs can alleviate the symptoms of UC, their use in medicine is severely constrained by drug resistance and associated ramifications, which include gastrointestinal reactions, dermatological problems, autoimmune disorders, and even cancer [8,9,10]. Thus, there is an urgent need to discover and develop affordable and reliable medicines.

The hypothesis that gut bacteria have a significant impact on the development of UC has gained credence in recent years as increasing evidence has accumulated [6]. Generally, biochemical and physical barriers prevent gut microflora (GM) from directly interacting with gut epithelial cells and mucosa. Once gut integrity is compromised, pathobionts can easily pass from the lumen to the lamina propria and induce the production of IL-1, IL-6, and IL-23, thus leading to the activation of pathogenic type 17 helper T-cells and eventually the recruitment of immune cells [11]. An imbalance between symbionts, commensals, and pathogens is believed to be involved in IBD. According to Machiels et al., Crohn’s patients had a lower abundance of butyrate-producing bacteria, such as *Faecalibacterium prausnitzii* and *Roseburia hominis* [12]. It has been reported that patients with IBD suffer from mucus depletion and exhibit decreased expression of tight junction proteins and microbiota translocation [13]. It may be effective in combating UC or IBD by restoring intestinal barrier function and microbiota homeostasis.

The Smilacaceae family member *S. glabra*, also known as Tu Fu Ling in China, has been extensively used as a food source and herbal remedy, with a variety of medicinal properties. *S. glabra* has also been used to cure and prevent a number of human illnesses, including metal poisoning, cancer, brucellosis, syphilis, inflammation, and acute and chronic nephritis, in many nations [11]. In the majority of investigations, small molecules, including flavonoids, monose-binding lectins, terpenoids, and polysaccharides, are thought to be the main bioactive elements in *S. glabra* [12,13]. However, it was shown that the polysaccharide from *S. glabra* has strong anti-inflammatory effects by reducing intestinal inflammation and blocking LPS-induced generation of TNF-, IL-6, and NO [14,15,16]. Additionally, *S. glabra* extracts markedly improved antioxidant enzyme activity, reduced excessive radical oxygen species (ROS), and suppressed the in vivo and in vitro production of IL-1β, IL-6, and TNF-α produced by lead (Pb) [17]. Considering the multiple pharmacological characteristics of the *S. glabra* extracts, this study suggests a promising treatment for UC. However, whether *S. glabra* polysaccharides can modulate the microbiota remains unknown in the dextran sodium sulfate (DSS)-induced inflammation model in mice.

To explore the mechanism of action of SGP-H in ulcerative colitis treatment, we prepared a mouse colitis model induced by DSS and analyzed the gut barrier function, inflammatory mechanisms, gut microbiota diversity, and their interrelation using data correlation analysis.

## 2. Materials and Methods 

### 2.1. Chemicals and Reagents

SGPs (batch number: GR-133–140421, purity ≥ 98% by high performance liquid chromatography (HPLC)) were obtained from GANG-RUN Biotechnology (Nanjing, China). DSS (molecular weight: 36,000–50,000 Da) was obtained from MP Biomedicals (Santa Ana, CA, USA). 5-Aminosalicylic acid (CAS:89-57-6 Molecular Formula: C7H7NO3; Molecular Weight: 153.14) was purchased from Sunflower Pharma (Jiamusi, China).

### 2.2. Experimental Design 

**Figure 1:**
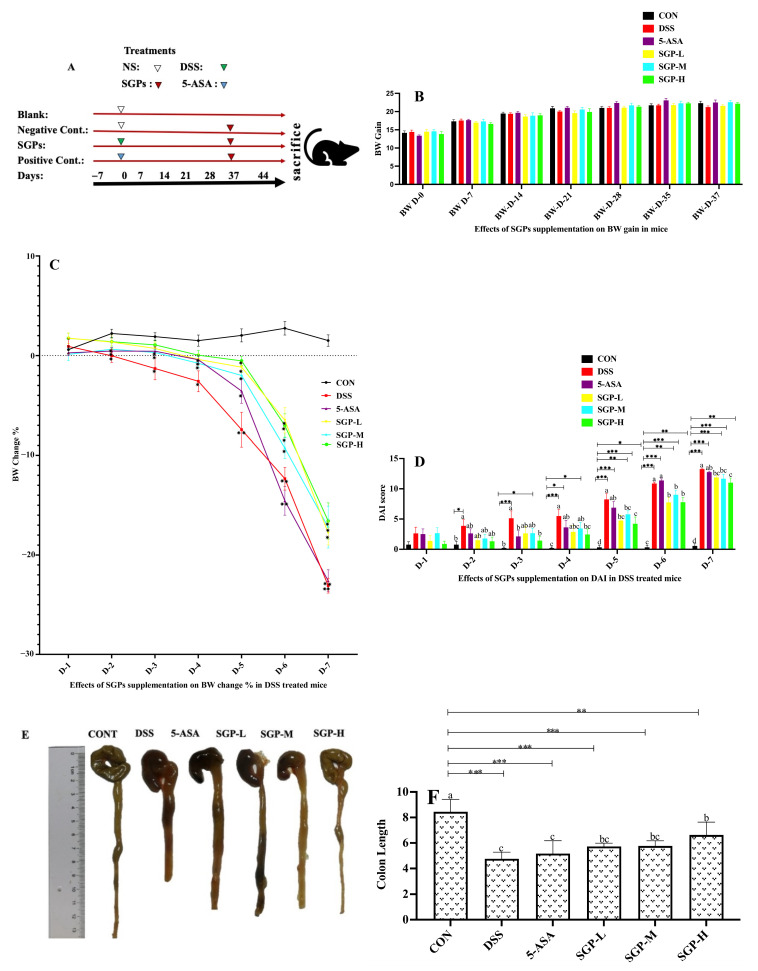
Effects of SGPs on mice with colitis induced by DSS. (**A**) Schematic representation of the experimental plan. (**B**) Body weight (BW) gain in SGPs supplemented mice. (**C**) % BW changes after DSS treatment. (**D**) DAI scores DSS treatments. (**E**) Colon size. (**F**) Average colon length in all groups. Data are shown as means ± SEM (*n* = 12 mice/group). The differences between the groups were. considered significant at * *p* < 0.05. In comparison to the control group, significance levels were denoted as * *p*  <  0.05, ** *p*  <  0.01, *** *p*  <  0.001. Different letters indicate significant difference between the respective groups while same letter indicates no significant difference.

72 C57BL/6 mice that were three weeks old were housed in groups of six mice per cage and given one week to acclimate before being used in the study. According to the plan shown in Figure 1A, all mice were randomly assigned to six separate groups, with six mice in each cage and 12 animals in each treatment group. The experimental procedures were performed according to the instructions of the Animal Ethics Committee of Henan Agricultural University, China (No. HNND2021100733). Group A was the blank control, and mice from groups B–F were treated with 4% DSS in drinking water on the 37th day for one week. Mice from groups D–F were supplemented with different doses of 800 mg/kg (H), 400 mg/kg (M), and 200 mg/kg (L) of *Smilax glabra* polysaccharides from day 0 to the 44th day. Mice from the positive control group (C) were gavaged with 5-aminosalicylic acid at 100 mg/kg from 0 to the 44th day. On the 28th day of the experiment, fresh sterile feces from all groups were collected for one week before and after DSS treatment and stored at −80 °C for future use. On the 44th day, the mice were euthanized by cervical dislocation; gut tissues were weighed, and blood, ileum, cecum, colon, spleen, and liver tissues were collected for further analysis. Fecal and blood samples were collected and sent to the company for metagenomics, and the rest of the experiments were performed in the laboratory.

### 2.3. Phenotype Evaluation of Enteritis in Mice

All mice underwent weekly general assessments, including body weight measurements and food and water intake measurements. Additionally, the disease activity index (DAI) of each mouse was rated using the same scoring system as previously discussed [18]. The score was the sum of parameters, including body weight loss (0 = none; 1 = 1–5%; 2 = 5–10%; 3 = 10–20%; 4 = 20%; 5 = >20%), stool consistency (0 = well-formed pellets; 1 = soft stool; 2 = loose stools; 3 = diarrhea; 4 = bloody diarrhea), and the presence or absence of fecal blood (0 = negative hemoccult test result; 2 = positive hemoccult test result; 4 = gross bleeding). Simultaneously, combined with pathological sections, the severity of enteritis was determined.

Body weight loss was determined by dividing the initial body weight (day 0) by the body weight on any given day after DSS treatment.

### 2.4. Histological Examination 

Slices of 4 μm thickness were made from paraffin-embedded ileum and colon tissues. Hematoxylin and Eosin (H & E) were used to stain the sections. Histological damage to the ileum and colon was observed under a light microscope, and histopathological scores were calculated using a previously mentioned grading system [19]. Briefly, the histological score was subdivided into the following categories: villus aspect (0 = normal, 1 = short, 2 = extremely short), villus top (0 = normal, 1 = damaged, 2 = severely damaged), epithelium (0 = normal, 1 = flattened, 2 = damaged, 3 = severely damaged), inflammation (0 = no infiltration, 1 = mild infiltration, 2 = severe infiltration), and crypts (0 = normal, 1 = mild crypt loss, 2 = severe crypt loss).

### 2.5. Alcian Blue Staining

Ileum and colon tissues were sectioned at 5 μm thickness, deparaffinized, and stained using an Alcian Blue staining kit (Xavier Bio Ltd., Fife, Scotland, UK) according to the manufacturer’s instructions. Goblet cells were observed and assessed in AB-stained and PAS-stained sections using a light microscope.

### 2.6. Apoptosis Detection Assay

Following the manufacturer’s recommendations, the In Situ TUNEL Apoptosis Detection Kit (batch number: MK1024, Wuhan Boster Biology Co., Ltd., Wuhan, China) was used to identify apoptotic cells in the colon. Briefly, the mice were immediately euthanized following the colitis experiment. Colon samples were collected and stained using the detection kit. DNA strand breaks were identified using terminal deoxynucleotidyl transferase (TdT). Fluorescence microscopy was used to detect fluorescein labels incorporated in the nucleotide polymers, and the frequency of TUNEL-positive cells was calculated. Five representative fields from each sample were counted to determine the total number of TUNEL-positive cells [20].

### 2.7. Immunohistochemical Analysis

Colonic slices that had been embedded in paraffin were deparaffinized, rehydrated, and washed with PBS for immunohistochemistry (IHC), according to the manufacturer’s instructions (Zhengzhou Nuohao Biology Science and Technology Co., Ltd., Zhengzhou, China). The sections were treated with 3% H_2_O_2_, microwaved for antigen retrieval, blocked with 3% bovine serum albumin (BSA), and then incubated with primary antibodies for an overnight period at 4 °C. Subsequently, the sections were stained with diaminobenzidine (DAB) and counterstained with hematoxylin after treatment with an HRP-labeled secondary antibody at room temperature for 50 min [21].

### 2.8. Real-Time qPCR

According to the manufacturer’s instructions, total RNA was extracted from approximately 50 mg of colon tissue using the TRIzol reagent, and its quantity was determined using spectrophotometry at 260 nm. The Superscript III first-strand synthesis method was used to generate complementary DNA (cDNA) from 2.5 μg of total RNA (Invitrogen, Carlsbad, CA, USA). QPCR was performed using SYBR Green reagents on a C1000 Touch PCR Thermal Cycler (BIO-RAD Laboratories, Shanghai, China). The primer sequences are listed in Supplementary File S1. mRNA levels were calculated using the 2^−ΔΔCT^ method and normalized to GAPDH levels.

### 2.9. Western Blot Analysis

Total tissue protein was extracted using RIPA lysis buffer and protease inhibitor phenylmethane sulfonyl fluoride (Merck Millipore, Burlington, MA, USA). A BCA protein assay kit (Beyotime Biotechnology, Shanghai, China) was used to measure the amount of protein in the sample, in accordance with the manufacturer’s recommendations. Equivalent amounts of protein (20 μg) were separated on an SDS PAGE gel and then transferred onto 0.45 μm PVDF membranes (Millipore, Billerica, MA, USA) according to standard protocols. Following established procedures, equivalent protein concentrations (20 μg) were separated on an SDS PAGE gel and then deposited onto 0.45 μm PVDF membranes from Millipore (Billerica, MA, USA). After being blocked with 5% milk in TBST buffer for 1 h at room temperature, the membranes were incubated with primary antibodies for an overnight period at 4 °C. Proteins were identified using ECL reagent following secondary antibody incubation for 1 h at room temperature (Millipore Billerica, MA, USA). Three independent replicates were performed for each [21].

### 2.10. Serum Cytokines Concentration

Blood was collected in tubes without anticoagulants. Blood samples were centrifuged at 3000 rpm for 10 min after getting coagulated to separate the serum, which was subsequently stored at 20 °C. An ELISA kit (Shanghai Mlbio Biotechnology, Shanghai, China) was used to measure the levels of IL-1β, IL-6, TNF-α, IL-10, and IL-20 cytokine proteins in the serum. Serum samples as sextuplets were diluted at a ratio of 1:50, loaded onto a 96-well plate covered with an antigen, then HPR conjugated and incubated for 60 min at 37 °C. The plates were washed with a 5× washing solution. In the dark, chromogen A and B solutions were added, and the plates were then incubated at 37 °C for 15 min. To cease the reaction process, a stop solution was added, and the plate was read at an optical density (OD) of 450 nm. The results were calculated as S/P = [(mean of test sample − mean of negative control)/(mean of positive control − mean of negative control)] ratio [22].

### 2.11. Microbiome Analysis

Mouse fecal samples were shipped on dry ice to Majorbio Bio-Pharm Technology Co., Ltd., Shanghai, China for microbiome analysis. Briefly, the E.Z.N.A.^®^ Soil DNA Kit was used to extract genomic DNA. DNA extracted from the sample was separated on an agarose gel, and the concentration and purity of the DNA were assessed using a NanoDrop 2000 UV-vis spectrophotometer (Thermo Scientific, Wilmington, NC, USA). Primer pairs 338F (5′-ACTCCTACGGGAGGCAGCAG-3′) and 806R (5′-GGACTACHVGGGTWTCTAAT-3′) were used to amplify the hypervariable region V3-V4 of the bacterial 16S rRNA gene using an ABI GeneAmp^®^ 9700 PCR thermocycler (ABI, Vernon, CA, USA). Purified amplicons were sequenced on an Illumina MiSeq sequencing platform (Illumina, San Diego, CA, USA) using PE300 chemicals (Majorbio company, Shanghai, China). Analysis of the 16S rRNA microbiome sequencing data was performed using the Majorbio Cloud Platform (www.majorbio.com).

### 2.12. Statistical Analysis

The number of experimental repetitions in all experiments was three. SPSS21 and Graph Pad Prism 7 were used to analyze the data. The results are presented as the mean ± standard error. Differences between groups were evaluated using one-way analysis of variance (ANOVA). Statistical significance was set at *p <* 0.05. In comparison to the control group, significance levels were denoted as * *p*  <  0.05, ** *p*  <  0.01, and *** *p*  <  0.001. Different letters indicate a significant difference between the respective groups, while the same letter indicates no significant difference. 

## 3. Results 

### 3.1. SGPs Ameliorated Colitis-Related Symptoms of DSS-Induced Acute Colitis

The effects of SGP supplementation on body weight gain were also observed. SGPs had no discernible influence on the weight gain of mice (Figure 1A). A growing body of research has revealed that UC is accompanied by weight loss [23]. Figure 1B shows the percentage of body weight loss in the mice after DSS treatment. Except for the CON group, all other groups showed a decrease in mouse weight. Compared with the CON group, the DSS and 5-ASA groups showed significantly higher weight loss. However, mice in the SGP-supplemented groups lost weight considerably more gradually than those in the DSS group, indicating that SGPs can relieve weight loss in mice receiving DSS.

DAI data typically reflect the severity of clinical symptoms developed in mice with DSS-induced colitis [24]. We estimated DAI using body weight loss, diarrhea, and bloody feces to determine whether SGPs mitigated the progression of UC [25]. As seen in Figure IC, mice supplemented with SGPs, particularly in the SGPH group, had considerably lower DAI scores than those treated with DSS. A significant reduction in colon length could be used to visually identify DSS-induced colitis. Notably, mice treated with DSS had shorter colons than the control mice, but mice supplemented with SGPs had longer colons than mice treated with DSS and 5-ASA. (Figure 1D,E). As shown in Figure 1E, both the low-dose and medium-dose groups of SGPs had significant differences in colon length from the DSS group, but the shortening of colon length was significantly attenuated in SGP-H-supplemented mice compared with the rest of the DSS-treated mice (*p* < 0.05), indicating that SGPs relieved colon disease. 

### 3.2. SGPs Ameliorated Histopathological Damage in Mice with DSS-Induced Colitis

**Figure 2:**
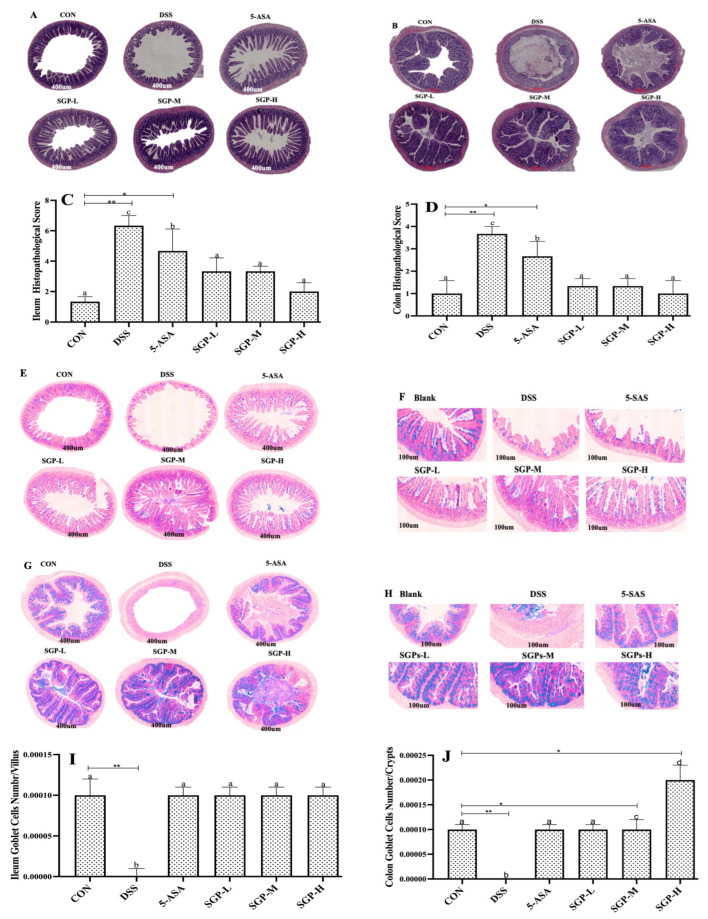

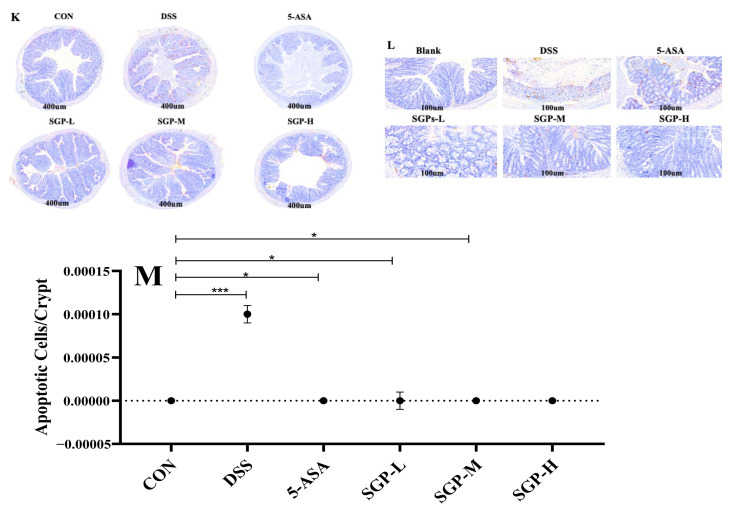
SGPs reserved gut histological injury triggered by DSS. (**A**,**B**) Characteristic photos of the ileum and colon tissues in H & E-stained pathological sections. Scale bar 400 μm. (**C**,**D**) Histopathological scores of ileum and colon tissues. (**E**,**F**) Representative images of Alcian blue/PAS-stained ileum sections. Scale bar, 400 μm (**E**) and 100 μm (**F**). (**G**,**H**) Representative images of Alcian blue/PAS-stained distal colon sections. Scale bar, 400 μm (**G**) and 100 μm (**H**). (**I**) Quantification of PAS-positive goblet cells per velus in the ileum tissue. (**J**) Quantification of PAS-positive goblet cells per crypt in the distal colon. (**K**,**L**) Apoptosis in the colon. Scale bar, 400 μm (**K**) and 100 μm (**L**). (**M**) the numbers of TUNEL-positive cells in the colon (at least 5 parts were evaluated for each sample). Data are shown as means ± SEM (*n* = 12 mice/group). The differences between the groups were considered significant at * *p* < 0.05. In comparison to the control group, significance levels were denoted as * *p*  <  0.05, ** *p*  <  0.01, *** *p*  <  0.001. Different letters indicate significant difference between the respective groups while same letter indicates no significant difference.

DSS has been shown to cause severe inflammation in both the small and large intestines [26,27,28]. Ileitis and ulcerative colitis were assessed using previously defined macroscopic damage scoring [19]. In contrast to CON mice (Figure 2A,B), DSS mice presented higher histopathological scores, wall thinning, extensive villus sloughing, loss of villi, and severe neutrophil infiltration. However, in the 5-ASA- and SGPs-supplemented mice, the number of intact villi increased, and neutrophil infiltration was reduced. Furthermore, the average colon morphological scores demonstrated that SGP supplementation considerably inhibited the increase in colon macroscopic scores caused by DSS as compared with the group that had received DSS only (Figure 2D). According to our histological analysis, DSS significantly increased inflammation, which was characterized by submucosal edema, reactive epithelial hyperplasia, substantial neutrophil infiltration, and nearly total crypt loss. Comparing therapy with SGPs to treatment with DSS, representative H&E histopathology data showed that the inflammatory response was significantly decreased by SGPs (Figure 2C). The blinded histological injury scores in the distal colon of the DSS+SGP-H group were significantly lower than those in the DSS, DSS+5-ASA, DSS+SGP-L, and DSS+SGP-M groups (Figure 2D).

Goblet cells are specialized epithelial cells that form the mucus layer by producing mucins and related proteins [28]. This layer protects the tissue from microbial penetration while preserving commensal balance and lessening the vulnerability to inflammatory bowel illnesses [29,30]. These results (Figure 2E–H) showed that SGP supplementation had a significant impact on goblet cell proliferation or secretion in the development of UC. However, compared with the control mice, DSS mice had a markedly decreased density of PAS-positive cells per villus or crypt. Collectively, these findings indicate that SGPs supplementation in the intestinal epithelium prevents goblet cell loss and preserves the integrity of the inner mucosa in DSS-induced colitis.

We evaluated cell death in the intestine using the TUNEL assay. We found that DSS induced cell death in colon crypts (Figure 2I). Quantitative analysis demonstrated that DSS enhanced the number of TUNEL-positive cells in the colon tissue compared with the control group, whereas SGP supplementation reduced the number of TUNEL-positive cells in DSS-induced colitis compared with the DSS group (Figure 2J). These findings indicate that SGP-H supplementation in the intestinal epithelium preserves the integrity of the inner mucus layer in DSS-induced colitis by lowering the number of apoptotic cells.

### 3.3. SGP-H Triggered the Differentiation of Tight Junction Proteines

**Figure 3:**
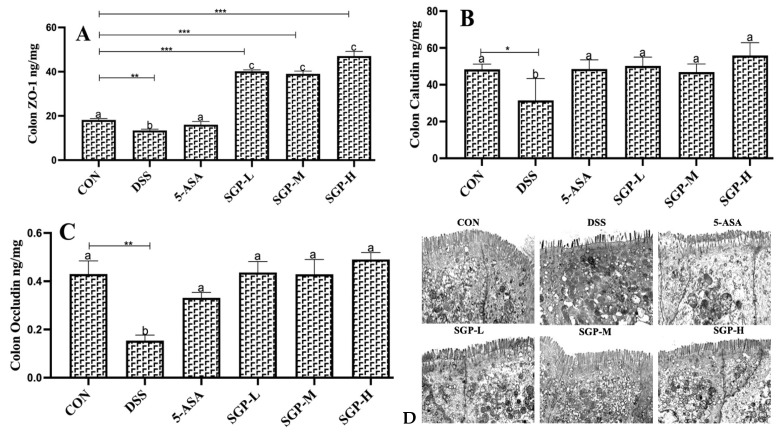
The level of ZO-1 (**A**), claudin (**B**) and occludin (**C**) in the mouse colon were assessed by ELISA. (**D**) TEM images present the effects of SGPs on structural defects in TJs upon DSS treatment. Data are shown as means ± SEM (*n* = 12 mice/group). The differences between the groups were considered significant at * *p* < 0.05. In comparison to the control group, significance levels were denoted as * *p*  <  0.05, ** *p*  <  0.01, *** *p*  <  0.001. Different letters indicate significant difference between the respective groups while same letter indicates no significant difference.

Multiple protein-based intercellular tight junctions (TJs) are essential for preserving epithelial integrity [31]. We found that DSS-induced colitis caused a reduction in the concentration of junctional proteins in the colon tissue (Figure 3A–C). SGPs block the effects of DSS-induced colitis. These findings indicated that SGPs significantly increased zonula (ZO-1), occludin, and claudin levels, indicating enhanced epithelial preservation and tight junction integrity.

Transmission electron microscopy (TEM) was used to analyze the structural integrity of tight junctions. The morphology of the enterocytes from CON and SGPs mice was similar; the microvilli were fully formed, and the cells were stuffed with mitochondria and vesicles (Figure 3D). The junctional complex was clearly apparent at the luminal side of cell-cell contact. Tight junctions were observed most apically, followed by adherens junctions and desmosomes from the apical to the basal side of the cell. The tight junctions in the colon of SGP-H mice were narrow, and kissing spots were observed, while at the kissing points, the intercellular spaces were completely locked. In contrast, the majority of tight junctions were poorly defined, “kissing points” were less frequent, and there was no complete obliteration of intercellular gaps in the colon of DSS mice. Our data were supported by morphometric analysis, which showed that DSS mice colons had significantly wider tight and adherent junctions than CON and SGP-H mice colons. However, in the colons of both CON and SGP-H mice, desmosomes were frequently identified with typical features, such as heavily stained plaques on the intercellular face of the membranes and a prominent midline.

Based on the above-mentoned ameliorative effects of SGPs against DSS-induced ulcerative colitis, our findings indicated that the emelioratant effect of SGP-H on gut inflammation was notably greater than that of all other groups. Consequently, SGP-H was chosen for subsequent analysis.

### 3.4. SGP-H Stimulates the Expression of MUC2 and MUC5a in Mice with DSS-Induced Acute Colitis

**Figure 4:**
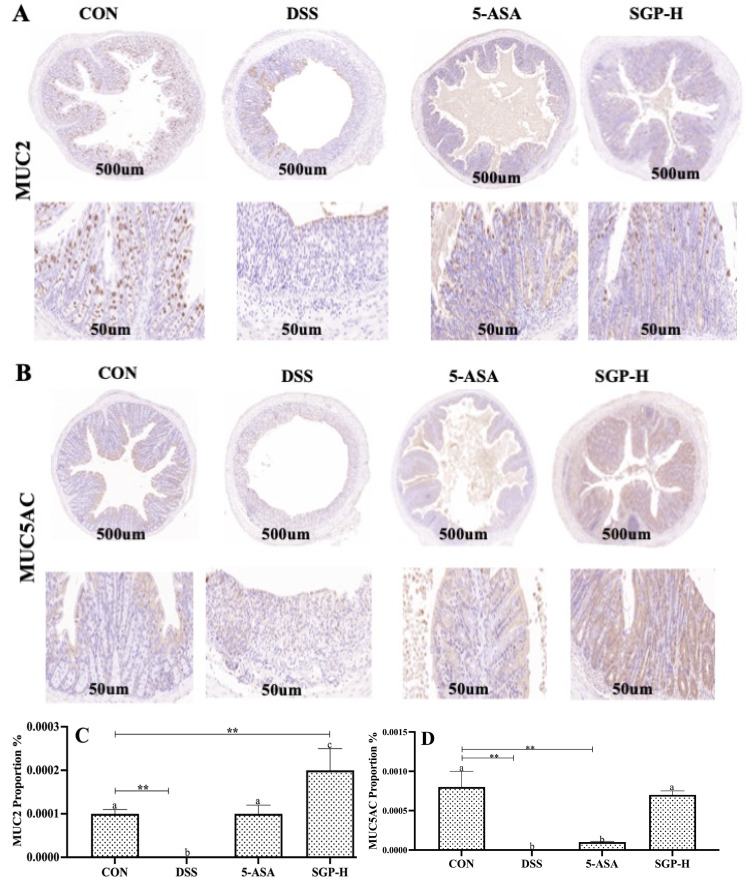
SGP-H improved the intestinal mucus layer barriers of mice with colitis. (**A**) Mouse colon MUC2 protein immunohistochemistry image Scale bar, 500 μm (upper panel) and 50 μm (lower panel). (**B**) Mouse colon MUC5AC protein immunohistochemistry image Scale bar, 500 μm (upper panel) and 50 μm (lower panel). Proportion of brown stained areas represent the abundance of mucin proteins. (**C**) MUC2 protein expression score in mouse colon. (**D**) MUC5AC protein expression score in mouse colon. The differences between the groups were considered significant. In comparison to the control group, significance levels were denoted as ** *p*  <  0.01. Different letters indicate significant difference between the respective groups while same letter indicates no significant difference.

Histological evaluation revealed that the colons of mice triggered by DSS were evidently damaged, and the number of goblet cells, which are specialized epithelial cells that secrete mucins and associated proteins to constitute the mucus layer, was remarkably decreased. To establish the protective effects of SGP-H against acute colitis, MUC2 and MUC5AC expression levels were evaluated after assessing the preservation of goblet cells in the SGP-H group. IHC analysis showed that the DSS group had reduced levels of MUC2 and MUC5AC protein expression. These pathological manifestations in the mouse colon were markedly reduced following SGP-H administration (Figure 4A,B). The results of the immunohistochemistry analysis demonstrated that SGP-H may prevent the DSS-induced decrease in mucoproteins MUC2 and MUC5AC. Based on these findings, we deduced that SGP-H significantly increases mucin secretion to provide a strong mucus-protective function.

### 3.5. SGP-H Modulate the Inflammatory Pathways and Downstream Pro-Inflammatory Cytokines Production

**Figure 5:**
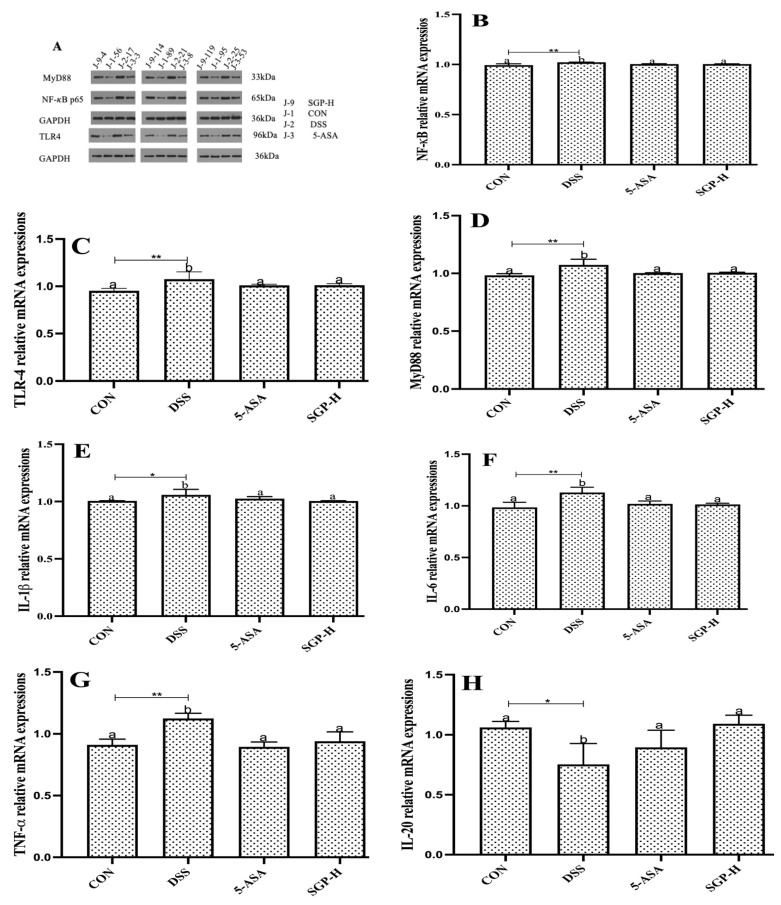

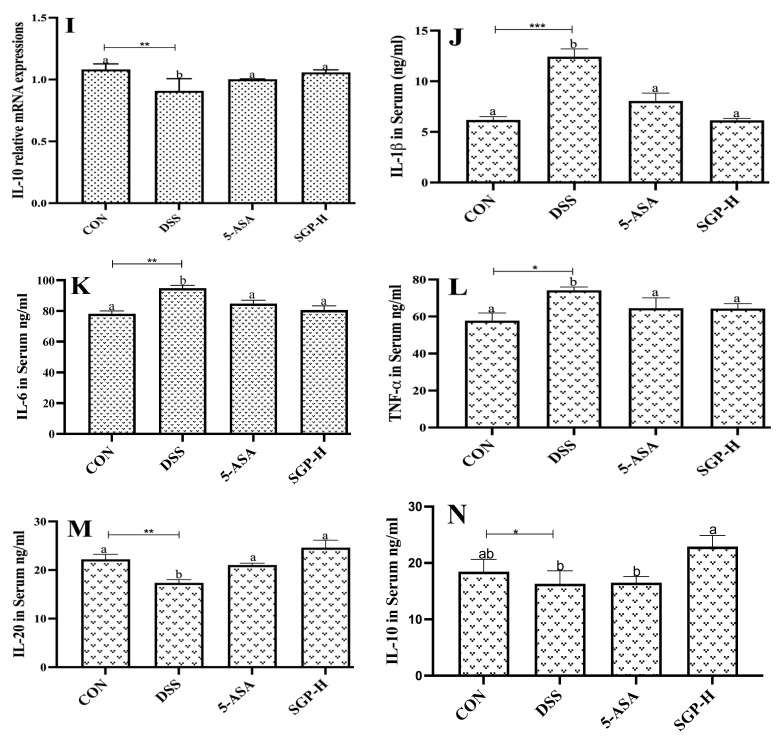
SGP-H blocked the inflammatory pathways and ameliorate the gut inflammation. (**A**) Western blotting of the expressions of the TLR4/MyD88/NF-κB signaling pathway. (**B**–**D**) Relative mRNA expressions of the TLR4/MyD88/NF-κB signaling pathway (**B**–**D**). Relative mRNA expression levels of inflammatory cytokines IL-1β (**E**), IL-6 (**F**) and TNF-α (**G**). Relative mRNA expression levels of anti-inflammatory cytokines IL-10 (**H**), IL-20 (**I**). Serum levels of inflammatory cytokines IL-1β (**J**), IL-6 (**K**) and TNF-α (**L**). Serum levels of anti-inflammatory cytokines IL-10 (**M**), IL-20 (**N**). The differences between the groups were considered significant at * *p* < 0.05. In comparison to the control group, significance levels were denoted as * *p*  <  0.05, ** *p*  <  0.01, *** *p*  <  0.001. Different letters indicate significant difference between the respective groups while same letter indicates no significant difference.

It is widely known that TLR4 and its downstream binding protein MyD88, which in turn activates NF-κB to trigger the production of inflammatory cytokines, are responsible for the DSS-induced increase in intestinal epithelial permeability [32]. As seen in Figure 5A, DSS-treated mice had significantly higher protein and relative mRNA expression levels (Figure 5B–D) of TLR4, MyD88, and NF-κB than the control group, whereas SGP-H treatment markedly decreased the expression of TLR4, MyD88, and NF-κB.

To confirm the ameliorative properties, we further evaluated the effect of SGP-H on pro-inflammatory cytokine concentrations, IL-1β, IL-6, and TNF-α, which are well-known markers of inflammation and play an important role in UC [33]. The results demonstrated that SGP-H treatment considerably decreased both relative mRNA expression (Figure 5E–G) and serum protein levels of IL-1β, IL-6, and TNF-α in the serum of mice treated with DSS (Figure 5J–L). Additionally, SGP-H can strengthen anti-inflammatory actions in mice with DSS-induced colitis. The relative mRNA expression of IL-20 and IL-10 (Figure 5H,I) and serum protein levels (Figure 5M,N) were considerably lower in DSS mice. Taken together, our findings indicate that SGP-H plays an anti-inflammatory role in enhancing gut barrier performance and reducing the pro-inflammatory reactions induced by DSS in mice.

### 3.6. SGP-H Improves Gut Microbiota Diversity in Colitis Mice

**Figure 6:**
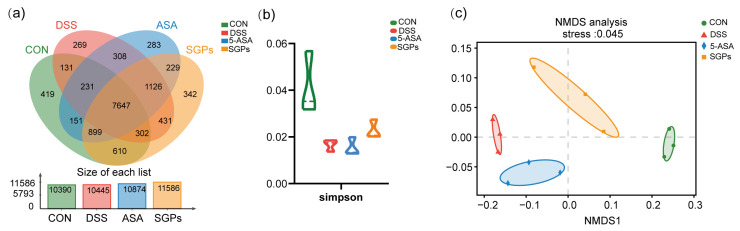
SGP-H enhanced the diversity of the gut microbiota in a mouse model of colitis. (**a**) The OTUs that were unique to each group are shown in the Venn diagram. (**b**) α-Diversity analysis: Simpson index at OTU level. (**c**) Beta-diversity Visualized Using NMDS Plot with Bray–Curtis Dissimilarity Distances.

Dysbiosis of the gut flora is one of the primary manifestations of IBD [6]. All samples were subjected to size filtering and quality control, and results at the OTU level (Figure 6a) demonstrated that the volume of sequencing information gleaned from each sample was sufficient. Venn diagram analysis at the OTU level later revealed an overlap between the four groupings: The Normal, DSS, DSS+5-ASA, and DSS+SGPH groups each had a total of 10,390, 10,445, 10,874, and 11,586 OTUs, respectively; 7647 OTUs overlapped across all groups when the cutoff criteria for sequence similarity was set at 97%. To evaluate alpha diversity, we computed Simpson indices (Figure 6b). Simpson indices were lower in the DSS group than in the CON group, but no statistically significant difference was observed between the groups.

Beta diversity was visualized using non-metric Multidimensional Scaling (NMDS) plots with Bray–Curtis dissimilarity distances (Figure 6c). NMDS plots on rank-order Bray–Curtis distances were used to assess the significance of bacterial composition between the groups. The results of the NMDS analysis showed that groups DSS and 5-ASA clustered closely, while CON and SGP-H clustered closely.

### 3.7. SGP-H Had Stronger Ability to Regulate the Gut Microbiota Composition in DSS-Induced Colitis

**Figure 7:**
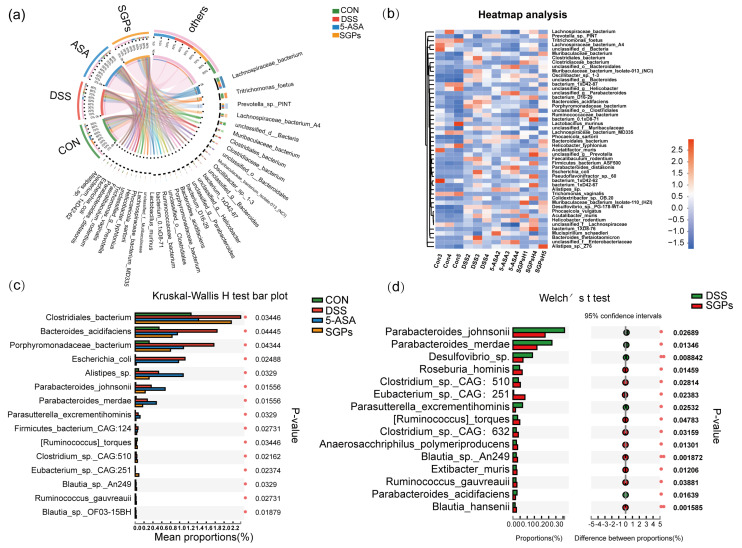
SGP-H influenced the species-level makeup of the gut microbiota in colitis mice. (**a**) Chord diagram showing species-level of the fecal microbiota. The upper half circle represents the four treatment groups, while the lower half circle represents the overall composition of fecal microbiota across all groups. (**b**) Community heatmap analysis of top 33 species. (**c**) Differential analysis among these four groups at the species level. (**d**) Differential analysis compared with DSS and SGP-H group at species level.

As SGP-H was able to control the diversity of the gut microbiota in mice, we next investigated whether SGP-H affected the composition of the gut microbiota. At the species level, community heatmap analysis represented 50 species (Figure 7a). Chord diagram Figure 7b shows the percentage levels of the top 33 species, and the most dominant species were *Lachnospiraceae bacterium*, *Prevotella*_sp_PINT, *Tritrichomonas_foetus* and *Lachnospiraceae_bacterium_A4*. The percentage abundance levels of these species in the different treatment groups are provided in Supplementary File S2. To further explore the impact of SGP-H on species in colitis mice, we analyzed species differences to compare the gut microbiota among the CON, DSS, SGP-H, and 5-ASA groups. We compared the gut microbiota between the CON, DSS, SGP-H, and 5-ASA groups to further understand the impact of SGP-H on species in colitis-induced mice. The Kruskal-Wallis H test bar plot at the species level (Figure 7c) shows that SGP-H treatment significantly decreased the relative abundance of *Bacteroides_acidifaciens*, *Porphyromonadaceae_bacterium*, *Escherichia_coli*, *Parasutterella excrementihominis*, and *Firmicutes bacterium_CAG: 124* in colitis mice, and significantly increased *Ruminococcaceae_torques*, *Clostridium_sp_CAG: 510*, *Eubacterium_sp_CAG: 251*, *Blautia_sp_An249*, *Ruminococcaceae_guvareauiii*, and *Blautia_sp_OF03_15BH*. In addition, the relative abundance of these species in the DSS group was substantially higher than that in the SGP-H group, according to Welch’s *t*-test at the species level (Figure 7d), including *Parabacteroides_johnsonii*, *Parabacteroides_merdae*, *Parabacteroides_acidifaciens*, *Desulfovibrio_sp*, *Parasutterella excrementihominis*, *Roseburia_hominis*, *Clostridium_sp_CAG: 510*, *Eubacterium_sp_CAG: 251*, *Blautia_sp_An249*, *Ruminococcaceae_torques*, *Clostridium_sp_CAG: 632*, *Anaerosacchariphilus_polymeriproducens*, *Blautia_sp_An249*, *Ruminococcaceae_guvareauiii*, *Extibacter muris*, and *Blautia_hansenii*. Collectively, these findings suggested that SGP-H, as compared with the DSS group, had a more profound effect on the gut microbiota composition in mice with DSS-induced colitis.

### 3.8. Functional Shift of the Mouse Colonic Microbiota following DSS Treatment in Response to SGP-H Supplementation

**Figure 8:**
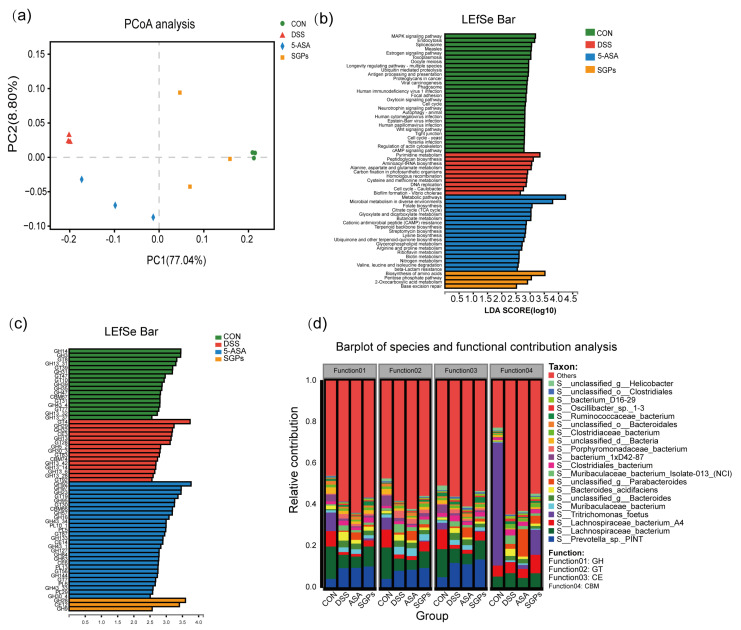
SGP-H supplementation modulated the function of gut microbs in the mice treated with DSS. (**a**) PCoA plot on the basis of KEGG pathways of reported microbial genes. (**b**) LEfSe analysis of differential enrichment of bacterial KEGG pathways (LDA > 2.5). (**c**) LEfSe analysis of differential enrichment of bacterial carbohydrate-active enzymes (CAZymes) (LDA > 3). (**d**) Functional contribution of top 6 differentially enriched CAZymes.

Metagenome sequencing was performed to investigate functional changes in the colonic microbiota of mice in response to DSS and SGP-H. Different functional profiles were found in the groups based on KEGG enzymes using the PCoA plot [analysis of similarity (ANOSIM) (Figure 8a)]. The LEfSe analysis revealed differential enrichment of various metabolic pathways. Functional pathways in the gut microbiota varied significantly in composition among various groups. We identified 60 pathways that were differentially abundant among the microbes of the CON, DSS, SGP-H, and 5-ASA groups. For example, the amino acid biosynthesis, pentose phosphate 2-oxocarboxylic acid metabolism, and base excision repair pathways were enriched in the SGP-H group (Figure 8b). We also observed the enrichment of several carbohydrate-active enzymes (CAZymes), such as glycoside hydrolase 28 (GH28), carbohydrate esterase (CE12), and glycoside hydrolases (GH9) in the SGP-H group (Figure 8c). In fact, *Lachnospiraceae bacterium* was the main contributor to these three CAZymes (Figure 8d).

### 3.9. SGP-H Controls the Consistency or Relationship between Microbiota, Inflammatory Cytokines, and Tight Junction Proteins

**Figure 9:**
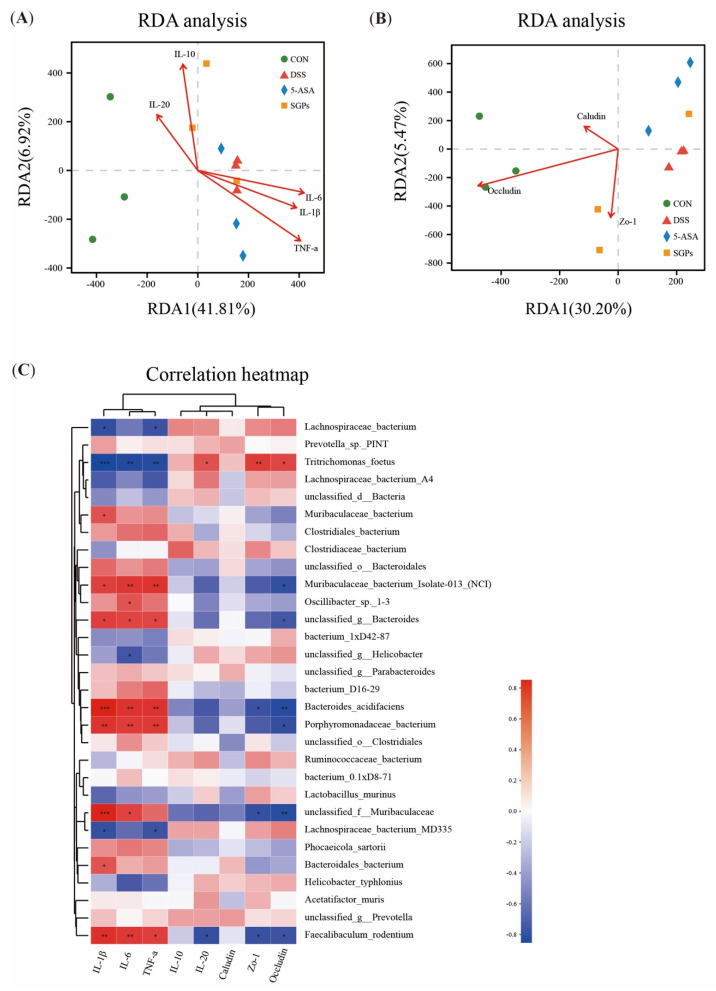
RDA analysis and Spearman’s correlation. (**A**) Correlation between inflammatory and ant-inflammatory cytokines and microbial flora structure displayed by distance-based redundancy analysis (db-RDA) (**B**) Correlation among tight junction proteins, and gut microbiota structure displayed by distance-based redundancy analysis (db-RDA). (**C**) Spearman’s correlation heatmap of inflammatory, ant-inflammatory cytokines, tight junction and gut microbiota. The differences between the groups were considered significant at * *p* < 0.05. In comparison to the control group, significance levels were denoted as * *p*  <  0.05, ** *p*  <  0.01, *** *p*  <  0.001.

The above findings suggest that SGP-H successfully controlled inflammatory cytokines, tight junction proteins, and gut microbiota and eased the inflammatory impairment in colitis mice. However, it is still unclear whether SGP-H controls the correlation between the gut microbiota, inflammatory cytokines, and tight junction proteins. Redundancy analysis (RDA) and Spearman’s correlation heatmap were used to examine their correlation in more detail. Based on the RDA/CCA analysis (Figure 8a,b), the levels of IL-10, 1 L-20, IL-6, IL-I β, TNF-α (Figure 9A), and tight junction proteins (Figure 9B) of colitis mice in the DSS group differed from those of the GM abundance in the CON and SGP-H groups at the OTU level, whereas the levels of these inflammatory cytokines and tight junction proteins in DSS were consistent with the gut microbiota abundance of the 5-ASA group. Spearman correlation heat map analysis found IL-6, IL-I β, and TNF-α (Figure 9C), to be positively correlated with *Faecalibaculum rodentium*, *Porphyromonadaceae_bacterium*, *Muribaculaceae bacterium isolate-013_(NCI)* and *Bacteroides_acidifacien*, especially 1 L-6 was positively corelated with Oscillibacter while all these three cytokines were negatively correlated with *Lachnospiraceae bacterium* and *Tritrichomonas_foetus*; IL-20 was positively correlated with *Tritrichomonas_foetus*, and negatively correlated with *Faecalibaculum rodentium*; ZO-1 was negatively correlated with *Faecalibaculum rodentium*, Unclassified_f_*Muribaculaceae*, and *Bacteroides_acidifaciens*; Occludin was negatively correlated with *Faecalibaculum rodentium*, Unclassified_f_*Muribaculaceae*, *Porphyromonadaceae_bacterium* and *Bacteroides_acidifaciens*, while ZO-1 and Occludin both these were positively correlated with *Tritrichomonas_foetus*.

## 4. Discussion

To effectively treat UC, a complex condition, there is a continuing quest for novel and effective medications with negligible or no side effects [5]. *S. glabra* is the rhizome of the Liliaceae plant [11] and has been shown to have several pharmacological benefits, including anti-infective [12,34], anticancer [35,36], anti-inflammatory [37], antioxidant [38], cardiovascular protection, etc. [39,40]. Previous studies have demonstrated promising anti-inflammatory properties of polysaccharides from *S. glabra* [14,15,16]. However, whether *S. glabra*-derived polysaccharides have any remedial effect on mucosal damage corresponding to IB has not been investigated. Here, we defined a new function for polysaccharides produced from *S. glabra* to reduce the intestinal inflammatory response to DSS in mice. Additionally, we verified a decrease in epithelial cell permeability, cytokine excretion, maintenance of tight junction integrity, and microbiota modulation in the intestine. 

The DSS model has a systemic inflammatory response that is coupled with bloody diarrhea, BW loss, and histopathologic alterations that resemble several clinical features of UC in humans [41]. We found that DSS led to a remarkable increase in the DAI score, a reduction in BW, and the presence of bloody stools, in addition to colonic shortness. Intriguingly, treatment with SGPs significantly improved these characteristics in mice with DSS-induced colitis, demonstrating that this polysaccharide may be a viable substitute to stop or slow the development of inflammatory diseases in the gut. Glucocorticoids, a type of traditional UC therapy, are currently used during the active period to maintain remission. However, with prolonged use, side effects, which may include peptic ulcers and impaired wound healing, continue to be a substantial issue [42]. In this way, SGP therapy not only reduced the severity of the disease in the DSS model, but earlier research has also shown that the polysaccharide from *S. glabra* has potent anti-inflammatory activity by reducing gut inflammation and inhibiting the LPS-induced production of pro-inflammatory cytokines [15], suggesting that SGPs may be a more effective option for treating UC.

To maintain homeostasis, the intestinal mucosal barrier serves as a vital first line of defense against the entry of infections and other objects into the colon [3]. Previous research has shown that natural polysaccharides can be used to alleviate IBD-related mucosal damage; however, the exact mechanism by which they achieve this is still not entirely understood [43]. In the current study, histological analysis showed that DSS caused inflammatory infiltration, goblet cell loss, apoptosis, and degradation of epithelial membranes, villi, and crypts in the ileum and colon. Conversely, pretreatment with SGPs substantially reduced gut inflammation and tissue damage, as seen by the prolonged colon, reduced histopathological score, increased goblet cell count, and decreased apoptotic cell count. Notably, SGP-H exerted superior effects on SGP-L and SGP-M. These findings imply that DSS-induced UC in mice may be successfully treated with SGP-H. This is consistent with earlier research on polysaccharides derived from other plants that could alleviate colitis in a DSS-induced mouse model [44,45].

Tight junctions (TJs) are intercellular complexes situated in the most apical portion of the junctional complex of intestinal epithelial cells and are essential for paracellular motility, cell-cell adhesion, and epithelial permeability [46]. TJs consist of transmembrane proteins (e.g., occludin, claudins, and junctional adhesion molecule A JAM-A) and intracellular membrane or scaffolding proteins (e.g., zonula occludens) and intracellular regulatory molecules (kinases and actin) [47]. Destruction of TJs may increase colonic permeability to toxic substances and dangerous microorganisms, causing diarrhea and an intestinal inflammatory response, which may promote the emergence and progression of UC [48]. Thus, therapeutic renovation of epithelial barrier function may play a crucial role in addressing the symptoms of UC. According to previous reports, polysaccharides reduce TJ disintegration and apoptosis in the colon epithelium of mice with DSS-induced colitis [49,50]. Likewise, in the current study, the downregulated tissue levels of TJ proteins, including ZO-1, occludin, claudin loss off microscopic AJ, TJ, DS, and the microvilli that constitute the brush border in DSS-induced colitis mice, were considerably improved by SGPs. Interestingly, SGP-H showed superior effects compared with the other groups. These findings showed that the safeguarding effect of SGP-H on DSS-induced colitis might be closely linked to increased transmembrane protein TJ protein production and improved epithelial TJs.

The intestinal tract epithelium is covered in mucus, which acts as the body’s first physical barrier and prevents microorganisms from penetrating the epithelial cells [51]. MUC2 and MUC5AC proteins secreted by goblet cells are the core components of colon mucus [52,53]. Mucus barrier malfunction caused by reduced mucin expression leads to UC [54]. Previous studies have shown that in DSS-treated mice, MUC2 and MUC5AC levels were reduced, which has been confirmed by previous research that DSS-treated mice had lower MUC2 and MUC5AC levels, which resulted in spontaneous colitis development [55,56]. Similarly, our IHC data demonstrated that treatment with DSS considerably decreased MUC2 and MUC5AC expression, whereas SGP-H relieved DSS-induced UC and promoted the expression of mucins such as MUC2 and MUC5AC. Our findings are in line with other studies [53,57], which may provide evidence that SGP-H could alleviate DSS-induced UC by inducing mucin production.

A growing body of evidence indicates that the TLR4, MyD88, and NF-κB pathways are the main regulators of the inflammatory response [58]. Increased mucosal permeability in UC patients allows toxic compounds and pathogenic bacteria to cross the intestinal wall, which activates TLR4 [59]. TLR4, a protein belonging to the family of Toll-like receptors, has been demonstrated to be upregulated in humans as well as animals with IBD [60,61]. The activation of TLR4 triggers the activation of MyD88, a chief adaptor molecule vital for TLR signaling. As a result, the downstream NF-κB signaling pathway is activated, which leads to the production of pro-inflammatory cytokines such as IL-6, IL-1β, and TNF-α, which aid in the progression of IBD [62]. Additionally, the impairment of epithelial barrier function and the collaboration between the mucosal immune system and gut microbiota are critically affected by NF-κB [63]. According to our findings, SGP-H pretreatment significantly deactivated TLR4, MyD88, and NF-κB pathways. Expression of NF-κB, TLR4, Myd88, and downstream molecules such as TNF-α, IL-6, and IL-1β was effectively reduced by SGP-H, which improved the secretion of anti-inflammatory cytokines including IL-20 and IL-10 in the colon tissue and serum of DSS-induced colitis mice. Notably, 5-ASA was outperformed by SGP-H in its ability to reduce inflammation. Our findings are in line with those of earlier studies showing that polysaccharides from various plants, including *Ganoderma lucidum* and *Gloiopeltis furcata*, may be able to treat colitis caused by DSS in a mouse model [64,65]. These findings suggest that the ability of SGP-H to treat DSS-induced colitis may be strongly linked to a reduction in inflammation by blocking the TLR4-MyD88-NF-κB signaling pathway.

As a metabolic “organ” with a key function in the absorption of sugars, fats, and xenobiotics, the gut microbiota is essential for ensuring human intestinal health [65]. Gut microflora plays a decisive role in the etiopathogenesis of UC [66]. As previously reported [67], DSS-induced colitis causes an imbalance in microflora and a decrease in gut microbial diversity. In the current study, SGP-H improved the microbiota composition richness and β-diversity compared with the model group that received DSS treatment. However, SGP-H did not have a significant effect on Î ± -diversity. Previous analysis revealed that IBD patients have dysbiosis of the gut flora and a higher abundance of pathogenic microbes *Clostridiales*, *Bacteroides_acidifacien*, *E. coli*, *Shigella*, *gamma-Amoeba*, and *Staphylococci* [68,69,70], and a lower abundance of *Lactobacillus*, *Bifidobacterium*, *Ruminococcus*, *Eubacterium*, and *Balutia* species [71,72,73,74]. We found that colitis mice had an intestinal flora imbalance, with a decreased abundance of *Ruminococcaceae_torques*, *Clostridium_sp_CAG: 510*, *Eubacterium_sp_CAG: 251*, *Blautia_sp_An249*, *Ruminococcaceae_guvareauiii*, and *Blautia_sp_OF03_15BH*, and enrichment of *Bacteroides_acidifaciens*, *Porphyromonadaceae_bacterium*, *Escherichia_coli*, *Parasutterella excrementihominis*, and *Firmicutes bacterium_CAG:124*. In addition, we observed that SGP-H treatment significantly decreased the relative abundance of pathobionts, including *Bacteroides_acidifaciens*, *Porphyromonadaceae_bacterium*, *Escherichia_coli*, *Parasutterella excrementihominis*, and *Firmicutes bacterium_CAG:124*, in colitis mice and significantly increased *Ruminococcaceae_torques*, *Clostridium_sp_CAG: 510*, *Eubacterium_sp_CAG: 251*, *Blautia_sp_An249*, *Ruminococcaceae_guvareauiii*, and *Blautia_sp_OF03_15BH*. Our findings are in line with those of other studies that have demonstrated that polysaccharides derived from different plants can reduce colitis in a DSS-induced animal model, which may be related to gut microbiota regulation [64,65].

Although the cause of IBD is still unknown, the prevailing opinion holds that dysbiosis-induced dysregulation of the immune system and an altered functioning mechanism are likely culprits [75,76]. Our goal was to understand the relationship between microbiome taxonomic levels and functional signatures. Metagenomic analysis has verified that the enrichment of microbial pathways, including amino acid biosynthesis, pentose phosphate 2-oxocarboxylic acid metabolism, and base excision repair pathways)and CAZymes such as GH28, E12, and GH9, are indicative of SGP-H-causing modifications to microbial operation. Additionally, it is intriguing to note that the current study CAZyme functions were strongly related to *Lachnospiraceae bacterium*, a well-known probiotic, and showed a remarkable increase in the SGP-H group, which is consistent with previous studies [77,78]. Notably, gut microflora plays a vital role in preserving intestinal homeostasis [79]. *Bacteroides_acidifacien*, *Oscillibacter*, *Porphyromonas*, and *Alistipes* endure pro-inflammatory characteristics, and over-enrichment can harm the intestinal mucosa [69,79,80,81,82]. In contrast, the *Lachnospiraceae* family promotes anti-inflammatory effects and intestinal mucosa healing [83]. We noticed that the therapeutic effect of SGP-H on UC was closely associated with the immunoregulatory effects of SGP-H, such as impeding inflammatory cytokine expression and fostering the differentiation of tight junction proteins. The mutual correlation between these effects and the microbiota was positive. The levels of IL-6, IL-Iβ, and TNF-α were positively correlated with *Faecalibaculum rodentium*, *Porphyromonadaceae_bacterium*, *Bacteroides_acidifacien*, and *Oscillibacter*, while all three cytokines were negatively correlated with *Lachnospiraceae bacterium* and *Tritrichomonas_foetus*; ZO-1 and Occludin were negatively correlated with *Faecalibaculum rodentium*, Unclassified_f_*Muribaculaceae*, *Porphyromonadaceae_bacterium* and *Bacteroides_acidifaciens*; ZO-1 and Occludin were positively correlated with *Tritrichomonas_foetus*. The changes we noticed in the taxonomic diversity and composition of the microbiome were largely reflective of functional capacity, which is consistent with previous findings [83]. Therefore, it is possible to state that SGP-H controls crosstalk between the innate immune system and intestinal physical barriers via the gut microbiota. According to the association, functional prediction, and evolution analyses, SGP-H improved the diversity of microbiota in DSS-induced colitis, which was related to the effects of SGP-H on inflammatory cytokine expression and tight junction protein differentiation.

Taken together, our findings suggest that SGP-H ameliorates mucosal barrier damage by increasing goblet cell secretion, tight junction protein differentiation, mucin production, and downregulating apoptotic cell number. Moreover, SGP-H demonstrated anti-inflammatory effects by blocking the NF-κB, TLR-4, and MyD88 pathways and regulating pro-inflammatory and anti-inflammatory cytokine production. SGP-H also regulated the composition of the gut microbiota and recovered the relative abundance of key bacteria, including *Roseburia _hominis*, *Clostridium_sp_CAG: 510*, *Eubacterium_sp_CAG: 251*, *Blautia_sp_An249*, *Ruminococcaceae_torques*, *Clostridium_sp_CAG: 632*, *Anaerosacchariphilus_polymeriproducens*, *Blautia_sp_An249*, *Ruminococcaceae_guvareauiii*, *Extibacter muris*, and *Blautia_hansenii*, and prevented the imbalance of intestinal flora in DSS-induced colitis in mice. In the future, we will continue to design a consortium consisting of key bacteria reported in the SGP-H group during our current research trial. We will also determine the mechanism of crosstalk between the gut microbiota and its metabolites at the cellular and genetic levels.

## Data Availability

The metagenomic sequencing data generated and analyzed in the present study are available in the NCBI Sequence Read Archive database under accession number PRJNA954974.

## Supplementary Materials

The following supporting information can be downloaded at: https://www.mdpi.com/article/10.3390/nu15194102/s1.
